# Supplementary material for: Plasmonic trimers designed as SERS-active chemical traps for subtyping of lung tumors
Source: Nat Commun. 2024 Jul 12;15:5855. doi: 10.1038/s41467-024-50321-0 (PMC11245553; doi:10.1038/s41467-024-50321-0)
Supplement: Supplementary file 1 — Supplementary Information [file 41467_2024_50321_MOESM1_ESM.pdf]

Supplementary Information for

Plasmonic trimers designed as SERS-active chemical traps for subtyping of lung tumors

Xing Zhao[1,5], Xiaojing Liu[2,5], Dexiang Chen[1,5], Guodong Shi[3], Guoqun Li[1], Xiao Tang[1], Xiangnan Zhu[1], Mingze Li[1], Lei Yao[1], Yunjia Wei[1], Wenzhe Song[1], Zixuan Sun[1], Xingce Fan[1], Zhixin Zhou[4], Teng Qiu[1,*] and Qi Hao[1,*]

[1]Key Laboratory of Quantum Materials and Devices of Ministry of Education, School of Physics, Southeast University, Nanjing 211189, P. R. China

[2]Department of Respiratory and Critical Care Medicine, the Affiliated Hospital of Qingdao University, Qingdao 266003, P. R. China

[3]Department of Thoracic Surgery, the Affiliated Hospital of Qingdao University, Qingdao 266003, P. R. China

[4]School of Chemistry and Chemical Engineering, Southeast University, Nanjing 211189, P. R. China

[5]These authors contributed equally: Xing Zhao, Xiaojing Liu, Dexiang Chen.

*Corresponding author. Email: tqiu@seu.edu.cn, qihao@seu.edu.cn;

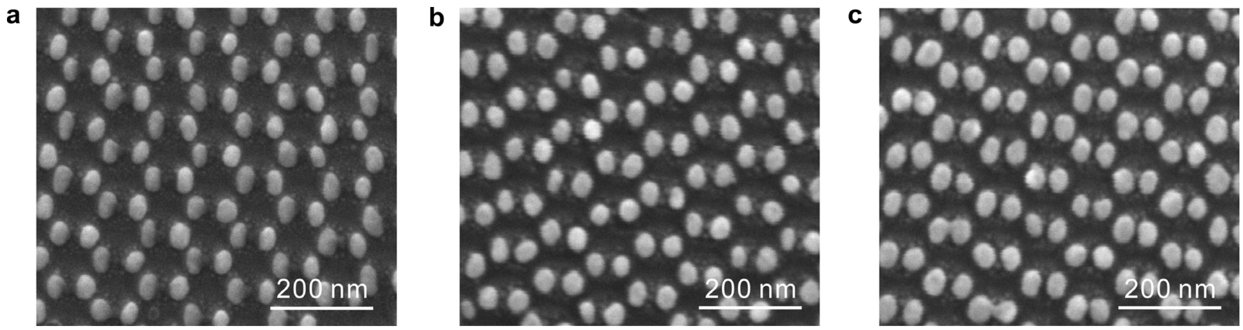

Supplementary Fig. 1. Modulation of the distance between the bilateral particles. a-c Au@Al_2O_3 dimers with gap distances of ≈ 30 nm (**a**), 20 nm (**b**) and 10 nm (**c**), respectively.The samples were fabricated by modulating the deposition angles during the angle-resolved shadow depositions.[1] The experiment was independently repeated at least three times with samples from different batchs, and the results were similar.

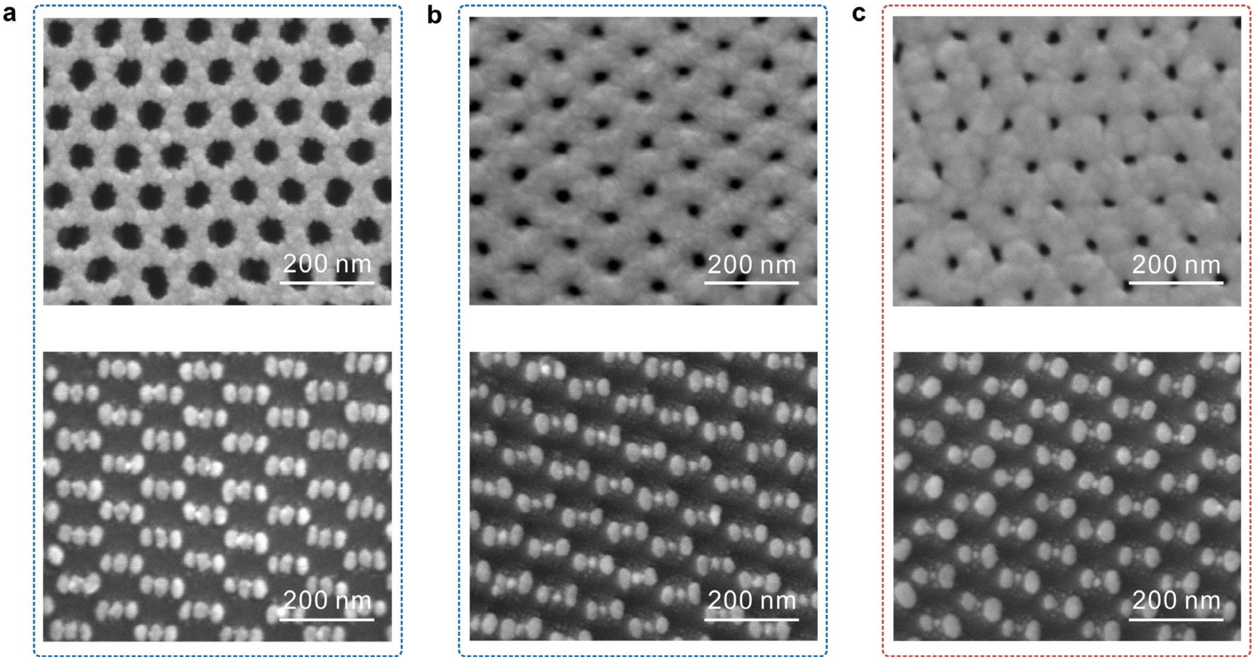

Supplementary Fig. 2. Modulation of the diamater of the trap particle. a-c SEM images of the AAO pores before (**a**) and after magnetron sputtering for 120 s (**b**) and 150 s (**c**), respectively, and the corresponding fabricated trimer arrays. The results reveal that the diameter of the trap particle can be modulated by adjusting the diameter of the AAO pores with magnetron sputtering. We employed the trimers presented in (c) in this paper because a smaller trap particle corresponds to a larger proportion of hotspots area relative to the entire Au surface exposed for molecular adsorption, which would be beneficial for the enrichment of probe molecules.

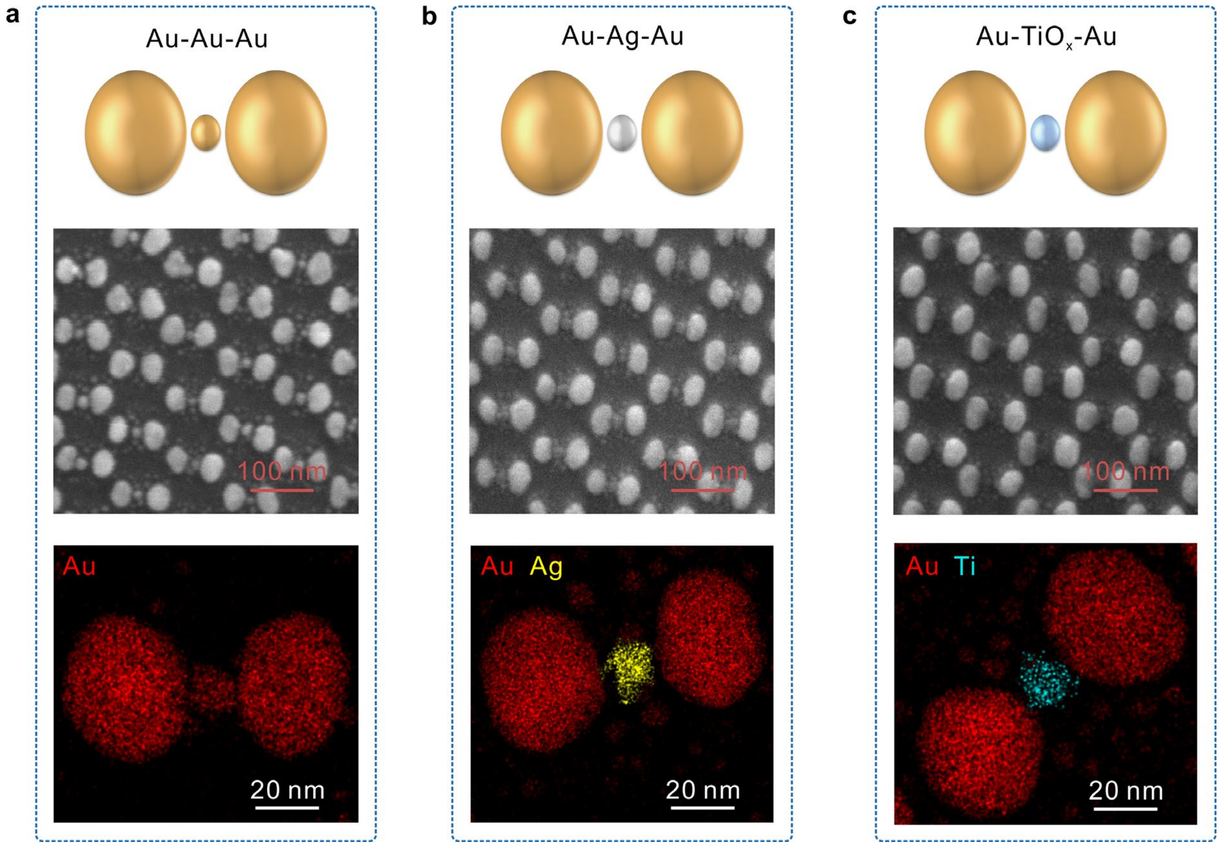

Supplementary Fig. 3. Characterizations of the plasmonic trimers with different components. **a**-**c**. Schematic diagrams, SEM images, and TEM images with corresponding element mappings of the Au-Au-Au (**a**), Au-Ag-Au (**b**) and Au-TiO_x-Au trimers (**c**), respectively. The TiO_x trap particle cannot be clearly identified under SEM due to the limited optical contrast between TiO_x and the SiO_2/Si substrate.

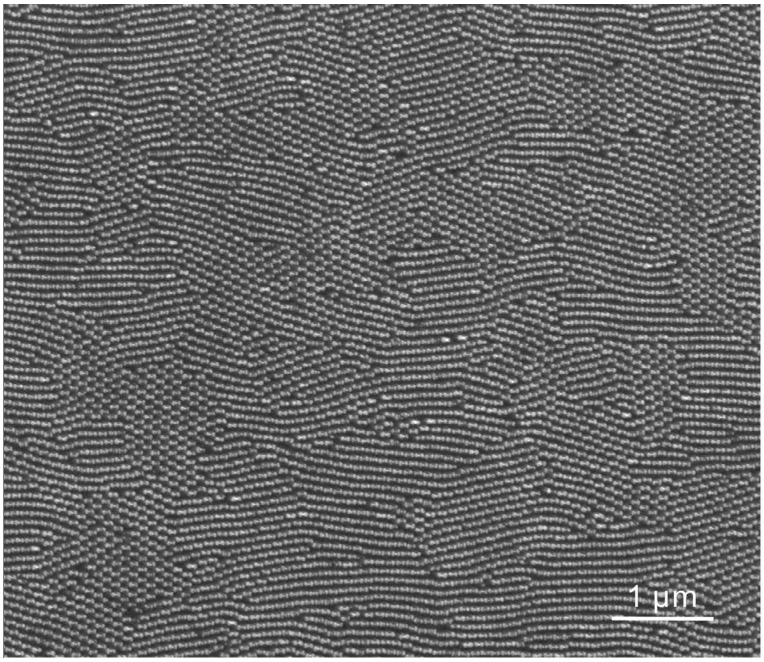

Supplementary Fig. 4. Large-scale SEM image of the Au@Al_2O_3-Au-Au@Al_2O_3 trimer arrays. The characterization was independently repeated dozens of times with samples from different batchs, and the results were similar.

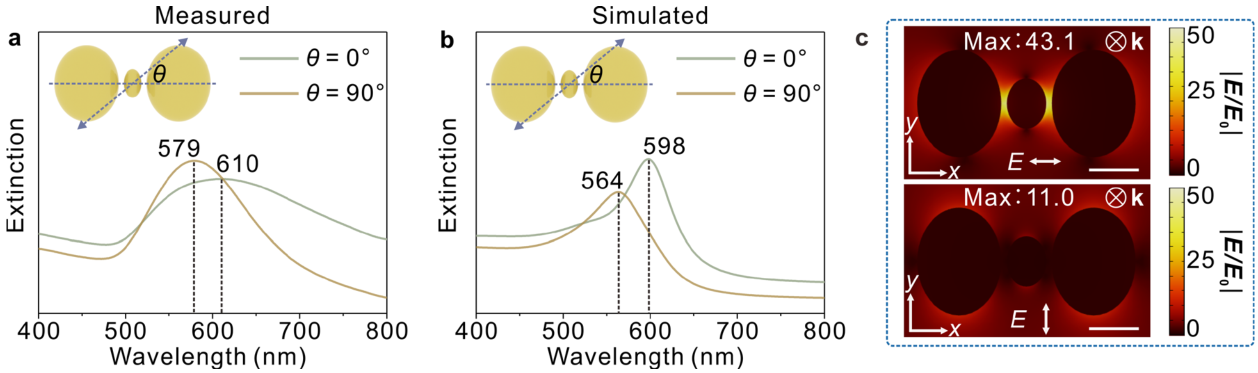

Supplementary Fig. 5. Polarized extinction spectra of the Au-Au-Au trimers. a, b Experimental and simulated polarized extinction spectra of the Au-Au-Au trimers, respectively. The mode located at ≈ 579 nm representing the long axis mode of the Au dimer ellipsoidal particle was observed under perpendicular excitation ($\theta = 90°$), while the modes suggesting the hybridized couplings between trimer particles were observed under horizontal excitation ($\theta = 0°$). There is an overall blueshift in spectra for the Au-Au-Au trimers compared with that for the Au@Al_2O_3-Au-Au@Al_2O_3 timers, arising from the changes in dielectric environment in the absence of the Al_2O_3 coating. **c** Local EM field distributions around the Au-Au-Au trimers under parallel and perpendicular polarized excitations at 785 nm. There is an overall decrease in EM intensity for the Au-Au-Au trimers compared from that for the Au@Al_2O_3-Au-Au@Al_2O_3 timers, and this is considered be to a secondary factor influencing the observed differences in SERS performance. The experimental parameters for the fabrication of the Au-Au-Au trimers are similar to those employed for Au@Al_2O_3-Au-Au@Al_2O_3, except for the absence of the Al_2O_3 coating. The simulation parameters were consistent with those described in the manuscript, and the results were in general agreement with experimental findings. The scale bars are 20 nm. Source data are provided as a Source Data file.

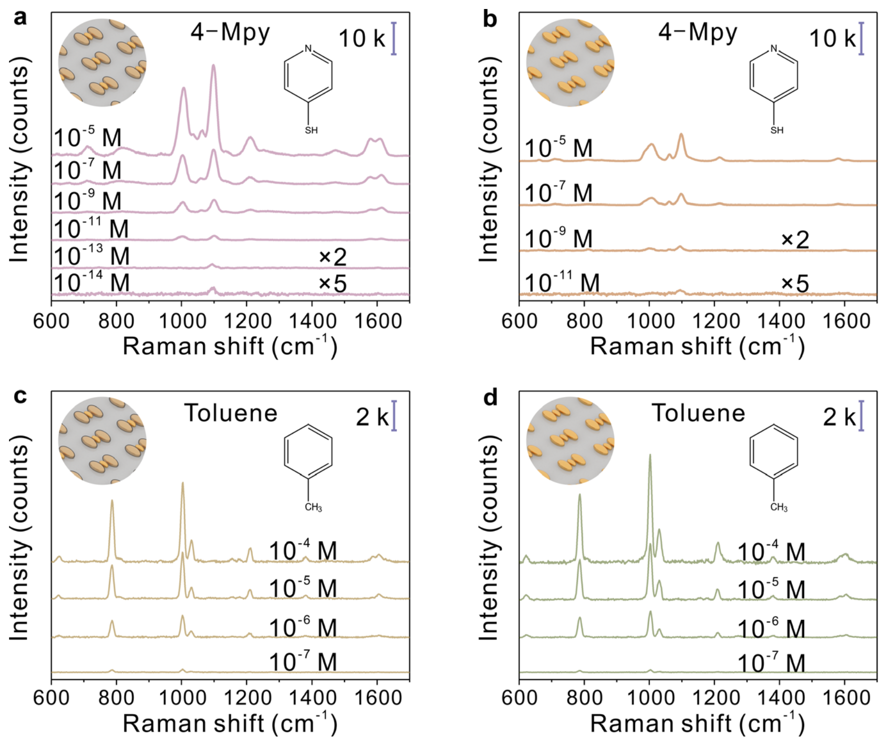

Supplementary Fig. 6. **SERS spectra of 4-Mpy and toluene on the Au@Al_2O_3-Au-Au@Al_2O_3 and Au-Au-Au trimers.** **a**, **b** SERS spectra of 4-Mpy from the Au@Al_2O_3-Au-Au@Al_2O_3 and Au-Au-Au trimers, respectively, at different concentrations. **c**, **d** SERS spectra of toluene from the Au@Al_2O_3-Au-Au@Al_2O_3 and Au-Au-Au trimers, respectively, at different concentrations (integral time 10 s). The results reveal that the Au@Al_2O_3-Au-Au@Al_2O_3 trimers has a higher specificity in identifying 4-Mpy while no significant differences were observed for toluene. Source data are provided as a Source Data file.

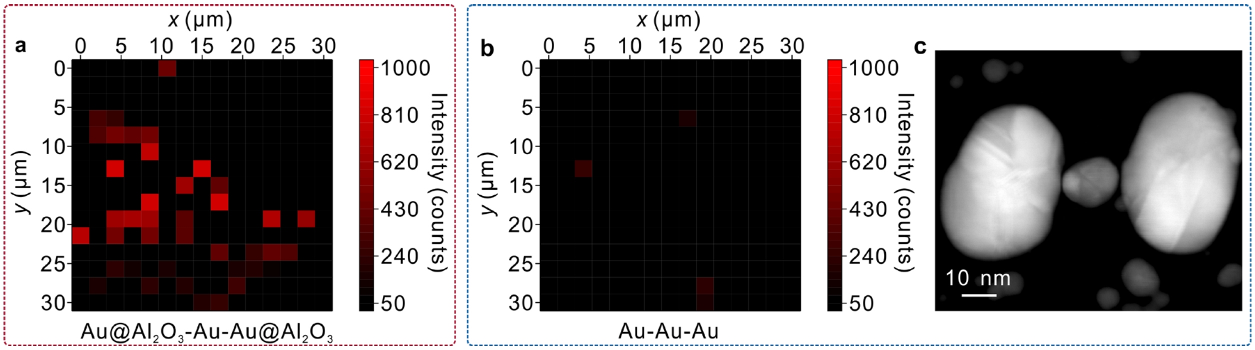

Supplementary Fig. 7. Comparative SERS performance between the Au@Al_2O_3-Au-Au@Al_2O_3 and Au-Au-Au trimers with a nanogap of ≈ 2 nm. a, b Raman intensity mappings of 4-Mpy at 1098 cm^{-1} from the Au@Al_2O_3-Au-Au@Al_2O_3 and Au-Au-Au trimers, respectively. (**c**) HAADF image of the Au-Au-Au trimers. Both the samples were immersed in a 10^{-11} M 4-Mpy solution (10 mL) for 4 h, followed by thorough rinsing with ethanol and drying with nitrogen gas. We employed the swift mapping mode to collect 225 spectra in a 30 × 30 μm^2 area (integral time 500 ms). Source data are provided as a Source Data file.

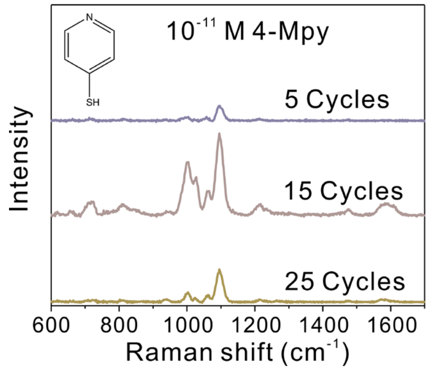

Supplementary Fig. 8. Optimizations of the alumina shell thicknesses for SERS investigations. The ALD coating is characterized by 0.1 nm per cycles. Considering the shape retention in ALD,[2,3] continuous alumina layer cannot be formed for 5 cycles, exposing gold atoms to the environment and diminishing the "trapping effects". While for the 25 cycles, a decrease in SERS was also observed. This is probably because that when the gap size is comparable to the molecules attempting to enter, molecules physically cannot fit through the gap due to steric hindrance. (integral time 10 s). Source data are provided as a Source Data file.

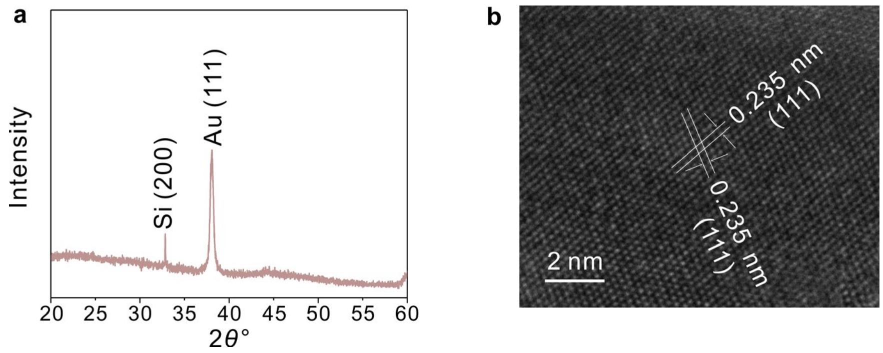

Supplementary Fig. 9. Characterizations of the gold particles deposited by e-beam evaporation. a XRD patterns of the Au@Al_2O_3-Au-Au@Al_2O_3 sample on silicon wafer substrate. The XRD patterns reveal a distinct peak at 38.2°, attributed to the (111) facets of fcc Au (JCPDS No. 04-0784), highlighting the crystallographic orientation of the material. **b** High-resolution transmission electron microscopy (HR-TEM) image of a gold nanoparticle produced by e-beam evaporation. The interplanar spacing of 0.235 nm was assigned to the (111) facet of face-centered (fcc) Au. These results indicate that the exposed facets of the gold nanoparticles preferred to be Au (111). Source data are provided as a Source Data file.

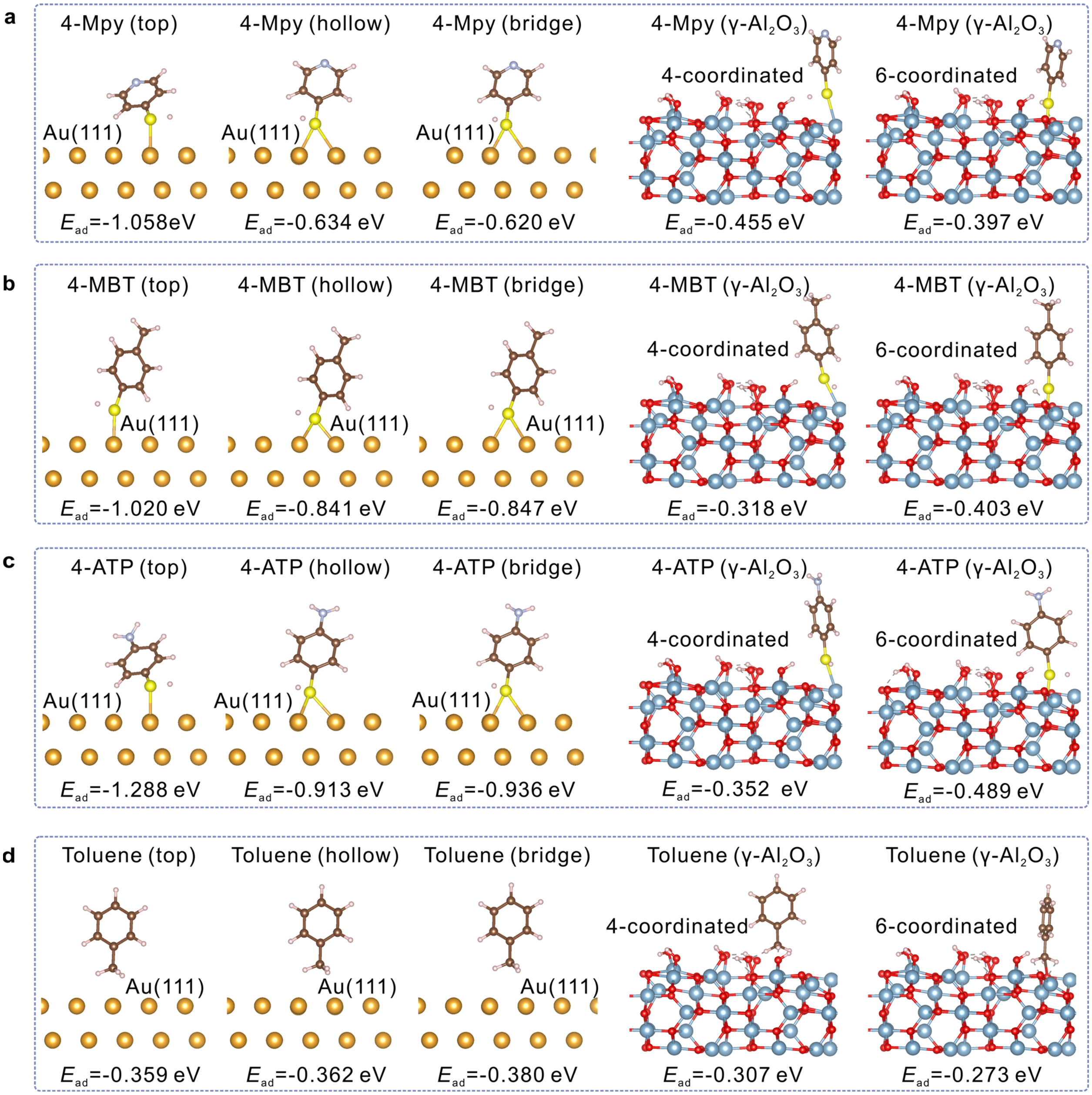

Supplementary Fig. 10. DFT calculation results for 4-Mpy, 4-MBT, 4-ATP and toluene. a Adsorption energy at different adsorption sites for 4-Mpy (a), 4-MBT (b), 4-ATP (c), and toluene (d), respectively, on Au(111) and γ-Al_2O_3(100). The results suggest a strong chemical adsorption of 4-Mpy, 4-MBT, and 4-ATP on Au (111), with the most stable state observed at the top site. In contrast, weak adsorption was observed for these molecules on γ-Al_2O_3(100). Toluene exhibits weak physical adsorption on both Au(111) and γ-Al_2O_3(100) with similar bonding affinities. Source data are provided as a Source Data file.

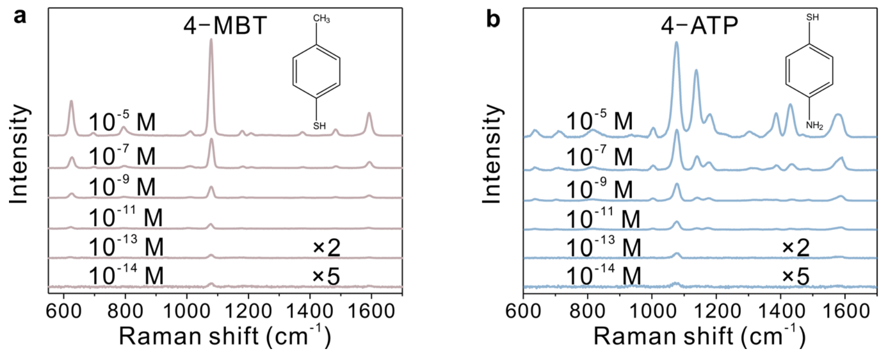

Supplementary Fig. 11. SERS spectra of 4-MBT (a) and 4-ATP (b) from the Au@Al_2O_3-Au-Au@Al_2O_3 trimers at different concentrations (integral time 10 s). Source data are provided as a Source Data file.

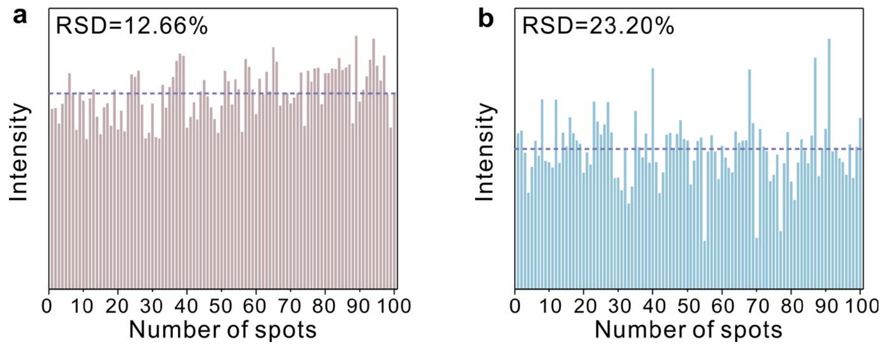

Supplementary Fig. 12. **SERS reproducibility results for the Au@Al_2O_3-Au-Au@Al_2O_3 and Au-Au-Au trimers. a, b** Peak intensities of 10^{-9} M 4-Mpy at 1098 cm^{-1} from the Au@Al_2O_3-Au-Au@Al_2O_3 and Au-Au-Au trimers, respectively. The results suggest that the signal flucturation was suppressed with the Au@Al_2O_3-Au-Au@Al_2O_3 trimers (integral time 10 s). The relative standard deviation (RSD) comes from n = 100 individual measurement points. Source data are provided as a Source Data file.

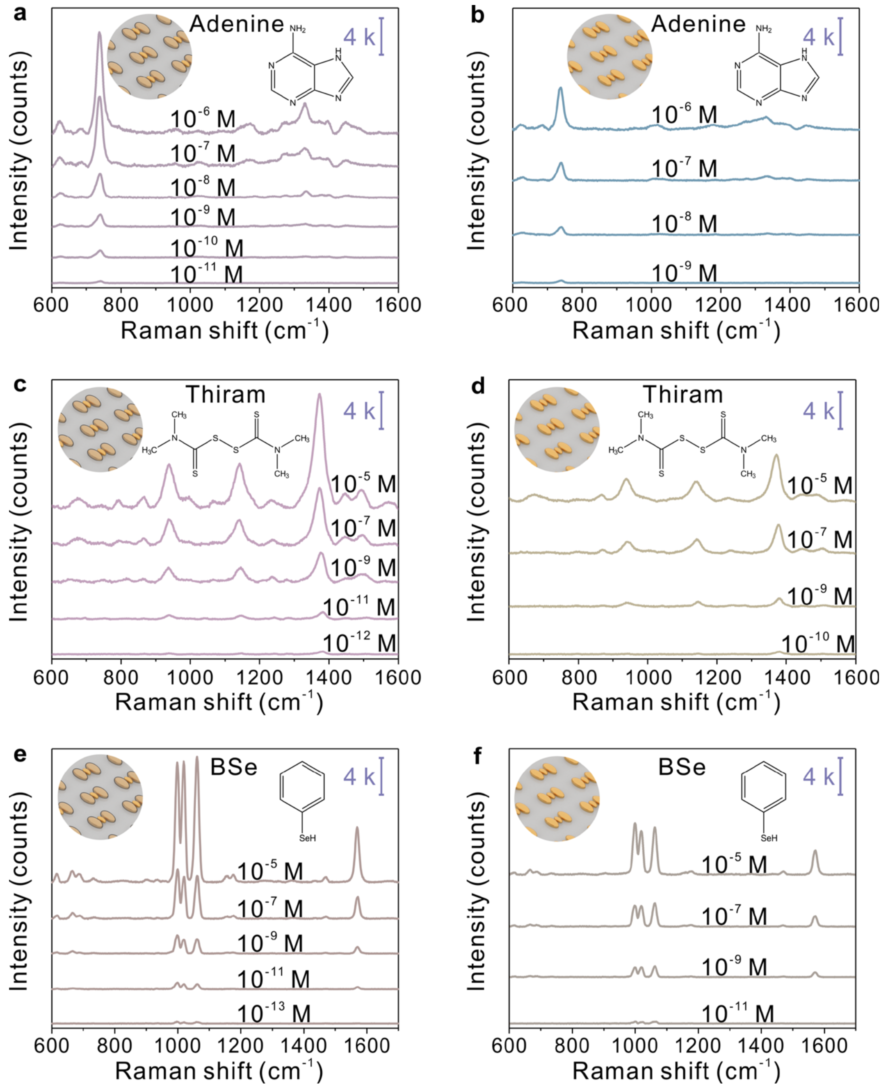

Supplementary Fig. 13. **Comparative SERS spectra of more probe molecules that selectivly adsorb on gold.** SERS spectra of adenine (**a**, **b**), thiram (**c**, **d**) and BSe (**e**, **f**), from the Au@Al_2O_3-Au-Au@Al_2O_3 and Au-Au-Au trimers, respectively (integral time 10 s). Improved SERS performance for all these analytes, specifically at low molecular concentrations, was observed for the Au@Al_2O_3-Au-Au@Al_2O_3 timers. The rationale for molecule selection lies in their high affinity towards the gold trap particles: Amino groups, with lone pair electrons on nitrogen atoms, can form strong coordinate bonds with gold atoms, while their interactions with alumina surfaces typically involve weaker hydrogen bonding with hydroxyl groups.[4–6] Disulfide molecules share similar characteristics with thiol analytes by establishing strong surface bonding with gold, while exhibiting inferior affinity to alumina.[7,8] Selenols form strong Au-Se bonds with gold surfaces, showing superior adherence compared to alumina.[9] Source data are provided as a Source Data file.

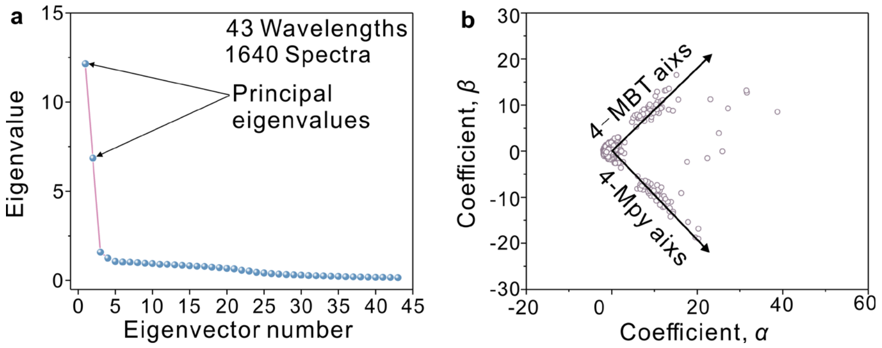

Supplementary Fig. 14. **MPCA algorithm for single-molecule SERS events. a** Eigenvalues obtained by MPCA of 1640 Raman spectra from random points. The characteristic peaks of 4-MBT at 1078 cm^{-1} and 4-Mpy at 1098 cm^{-1} were selected as typical peaks for analysis. **b** Covariance matrix coefficients (α and β) distributions obtained by MPCA analysis, and the principal component coordinate axes are used to describe the data. The events containing signal of only 4-MBT or 4-Mpy are located near x or y coordinate axis, respectively, while mixed events are located between the two coordinate axes, and blank events are located near the origin point. Source data are provided as a Source Data file.

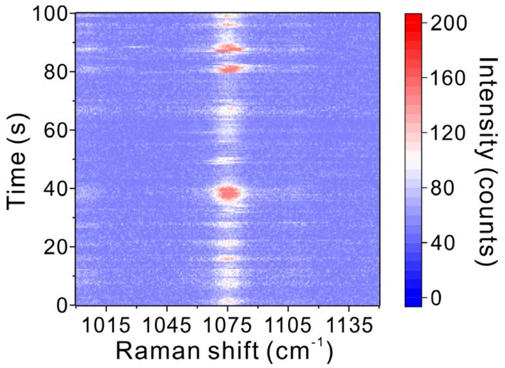

Supplementary Fig. 15. Real-time Raman dynamics of a single-molecule 4-MBT event. The single-molecule SERS event of 4-MBT sorted out by the bi-analyte method. The results reveal an obvious blinking, indicating a high probability of a single-molecule event. Besides, the wandering phenomenon is not observed. We surmise that this may arise from the simplicity of the molecule and the strong bonding between the molecule and substrate (integral time 500 ms). Source data are provided as a Source Data file.

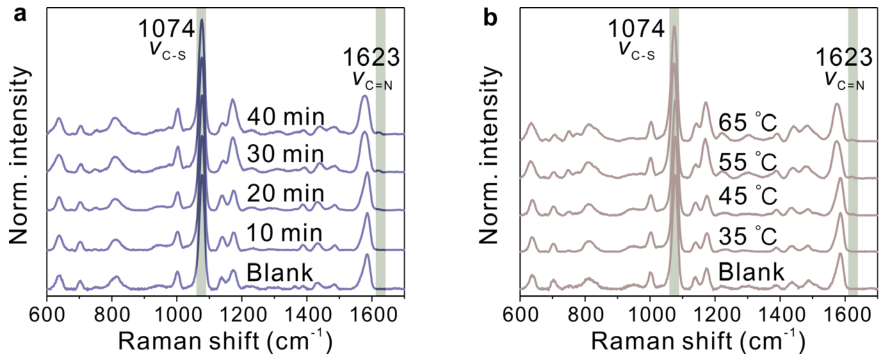

Supplementary Fig. 16. Experimental optimizations for the detection of BA molecules. a, b Normalized SERS spectra of 10^{-9} M BA molecules with different reaction times at 55 °C (**a**) and at different reaction temperatures for 30 min (**b**). The results reveal that the adsorption reaches near-saturation levels at 55 °C for 30 min (integral time 10 s). Source data are provided as a Source Data file.

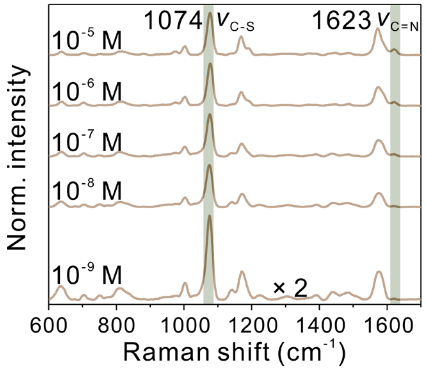

Supplementary Fig. 17. **SERS spectra of BA at different concentrations.** Normalized SERS spectra from the 4-ATP modified Au@Al_2O_3-Au-Au@Al_2O_3 trimers after the collection of BA molecules at different concentrations. The trimer samples were placed on top of the solutions at 55 °C for 30 min for molecular collection (integral time 10 s). Source data are provided as a Source Data file.

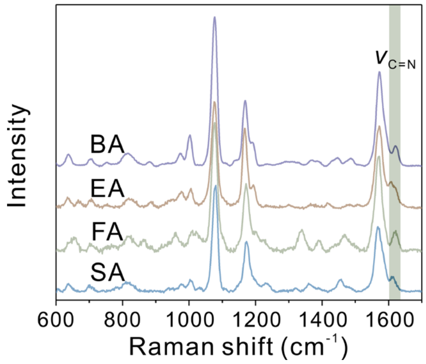

Supplementary Fig. 18. SERS spectra of other representative aldehyde molecules. Raman spectra of the 4-ATP modified Au@Al_2O_3-Au-Au@Al_2O_3 trimer samples after reacting with benzaldehyde (BA), 4-ethylbenzaldehyde (EA), 2-furaldehyde (FA) and salicylaldehyde (SA) at a concentration of 10^{-5} M. The C＝N vibration mode at 1623 cm^{-1} was observed for all the samples, suggesting the reaction between aldehyde molecules and 4-ATP (integral time 10 s). Source data are provided as a Source Data file.

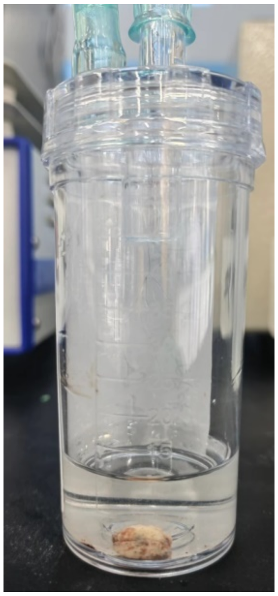

Supplementary Fig. 19. Optical photo of the lung cancer tissue. The SERS chip was subsequently immersed in the solution to collect the Raman signal of aldehyde molecules.

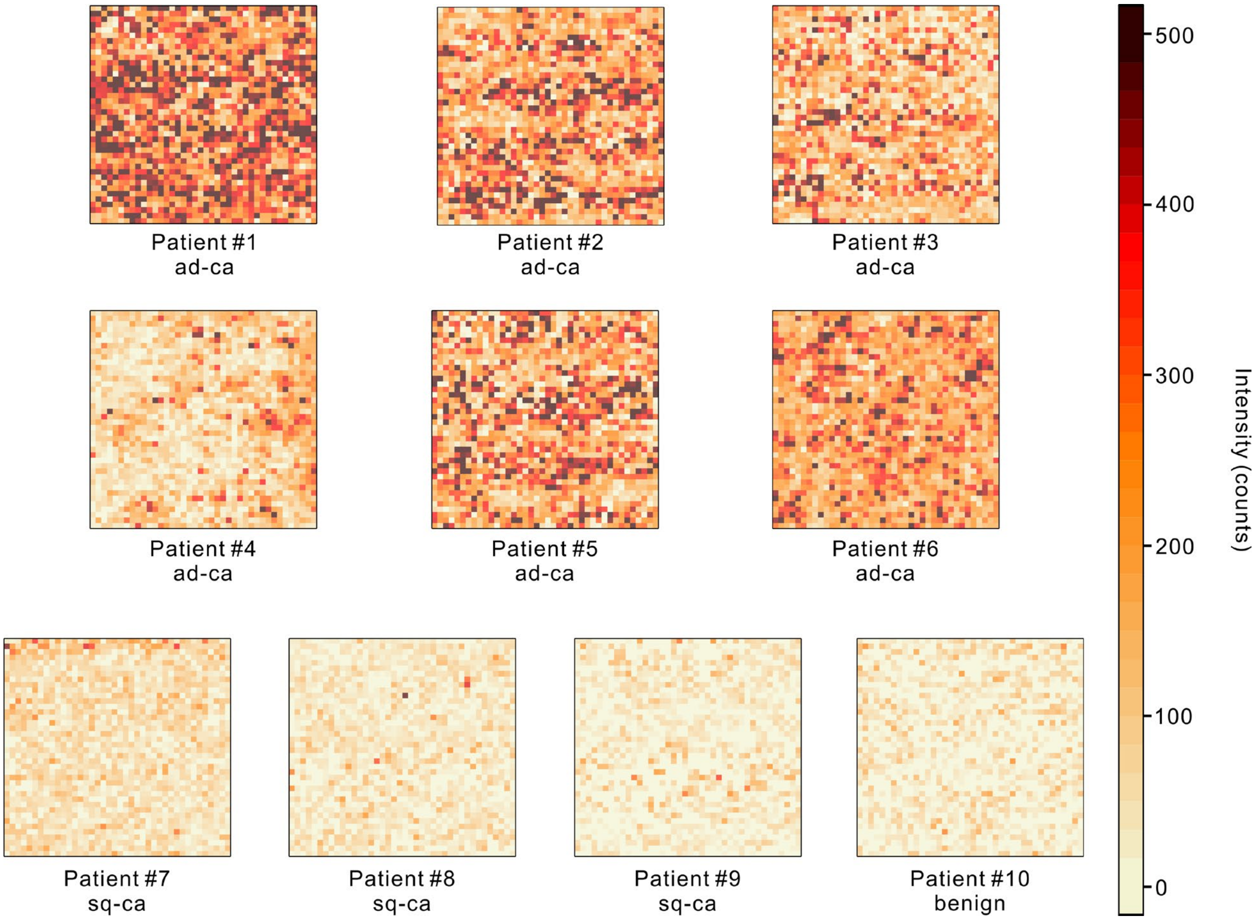

Supplementary Fig. 20. Raman mappings of samples with different tumor subtypes. It is observed that though the intensity of Raman mapping fluctuated, the signal of the ad-ca samples was stronger than that of the sq-ca samples. Besides, no significant differences in signal intensity were observed between the sq-ca samples and benign tumor sample (integral time 10 s). Source data are provided as a Source Data file.

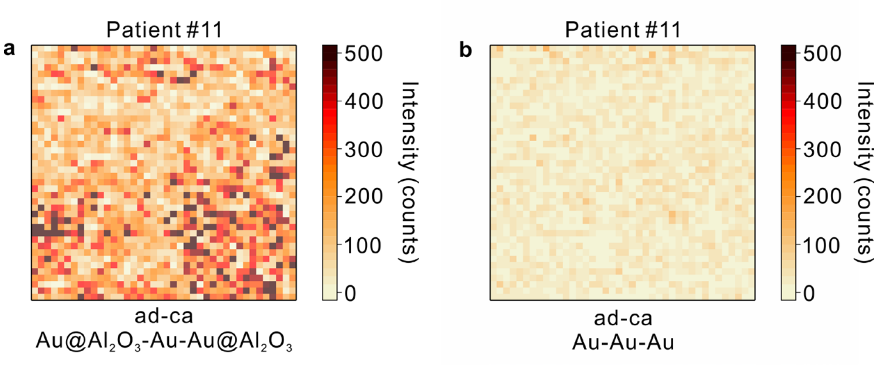

Supplementary Fig. 21. Comparative SERS mappings from the Au@Al_2O_3-Au-Au@Al_2O_3 and Au-Au-Au trimers. a, b Raman mappings at 1623 cm^{-1} of an ad-ca sample (patient #11) from the Au@Al_2O_3-Au-Au@Al_2O_3 (**a**) and Au-Au-Au (**b**) trimer arrays. Both the samples had been functionalized with 4-ATP molecules, and immersed into the solution containing target tissue simultaneously during the preparations (integral time 10 s). Source data are provided as a Source Data file.

Supplementary Table 1. Information on the tumors of lung cancer patients.

ID	Subtypes	Tumor Node Metastasis (TNM) Stage	Tumor Size
Patient #1	ad-ca	pT2aN0M0; IB	3.3*1.4*1.3 cm^3
Patient #2	ad-ca	pT2aN1M0; IIB	3.2*2.5*2.0 cm^3
Patient #3	ad-ca	pT2aN0M0; IB	3.5*2.2*1.9 cm^3
Patient #4	ad-ca	pT1aN0M0; IA1	1.8*1.0 *1.0 cm^3
Patient #5	ad-ca	pT1cN2M0; IIIA	2.7*2.5*2.2 cm^3
Patient #6	ad-ca	pT1aN0M0; IA1	2.0*1.0*1.0 cm^3
Patient #7	sq-ca	pT2aN0M0; IB	3.3*2.0*1.6 cm^3
Patient #8	sq-ca	pT2aN0M0; IB	4.0*3.0*3.0 cm^3
Patient #9	sq-ca	pT3N0M0; IIB	5.5*5.0*5.0 cm^3
Patient #10	benign tumor	N/A	2.1*1.5*1.1 cm^3
Patient #11	ad-ca	pT1aN0M0; IA2	1.8*1.6*1.0 cm^3

Supplementary References

1. Hao, Q. et al. Controlled Patterning of Plasmonic Dimers by Using an Ultrathin Nanoporous Alumina Membrane as a Shadow Mask. *ACS Appl. Mater. Interfaces* **9**, 36199–36205 (2017).
2. Zhang, X., Zhao, J., Whitney, A. V., Elam, J. W. & Van Duyne, R. P. Ultrastable Substrates for Surface-Enhanced Raman Spectroscopy: Al_2O_3 Overlayers Fabricated by Atomic Layer Deposition Yield Improved Anthrax Biomarker Detection. *J. Am. Chem. Soc.* **128**, 10304–10309 (2006).
3. Li, J. F. et al. Shell-isolated nanoparticle-enhanced Raman spectroscopy. *Nature* **464**, 392–395 (2010).
4. Lyu, Y. et al. The Interaction of Amines with Gold Nanoparticles. *Adv. Mater.* **36**, 2211624 (2024).
5. Östblom, M., Liedberg, B., Demers, L. M. & Mirkin, C. A. On the Structure and Desorption Dynamics of DNA Bases Adsorbed on Gold: A Temperature-Programmed Study. *J. Phys. Chem. B* **109**, 15150–15160 (2005).
6. Lee, S.-H. et al. Photooxidation of Amine-Terminated Self-Assembled Monolayers on Gold. *J. Phys. Chem. C* **114**, 10512–10519 (2010).
7. Nuzzo, R. G., Zegarski, B. R. & Dubois, L. H. Fundamental studies of the chemisorption of organosulfur compounds on gold(111). Implications for molecular self-assembly on gold surfaces. *J. Am. Chem. Soc.* **109**, 733–740 (1987).
8. Nuzzo, R. G. & Allara, D. L. Adsorption of bifunctional organic disulfides on gold surfaces. *J. Am. Chem. Soc.* **105**, 4481–4483 (1983).
9. Huang, F. K., Horton, R. C., Myles, D. C. & Garrell, R. L. Selenolates as Alternatives to Thiolates for Self-Assembled Monolayers: A SERS Study. *Langmuir* **14**, 4802–4808 (1998).
